# Fabrication of polycaprolactone electrospun fibres with retinyl acetate for antioxidant delivery in a ROS-mimicking environment

**DOI:** 10.3389/fbioe.2023.1233801

**Published:** 2023-08-15

**Authors:** Lorna Westwood, Elaine Emmerson, Anthony Callanan

**Affiliations:** ^1^ School of Engineering, Institute for Bioengineering, University of Edinburgh, Edinburgh, United Kingdom; ^2^ The Centre for Regenerative Medicine, Institute for Regeneration and Repair, University of Edinburgh, Edinburgh, United Kingdom

**Keywords:** tissue engineering, antioxidant, drug delivery, tumor microenvironment, repair, regeneration, salivary gland, electrospinning

## Abstract

**Background:** Increased cancer rates denote that one in two people will be diagnosed with cancer in their lifetime. Over 60% of cancer patients receive radiotherapy, either as a stand-alone treatment or in combination with other treatments such as chemotherapy and surgery. Whilst radiotherapy is effective in destroying cancer cells, it also causes subsequent damage to healthy cells and surrounding tissue due to alterations in the tumor microenvironment and an increase in reactive oxygen species (ROS). This can cause extensive damage that impairs tissue function, and the likelihood of tissue regeneration and restoration of function is significantly reduced as new healthy cells cannot survive in the damaged environment. In the treatment of head and neck cancers, radiotherapy can cause salivary gland dysfunction. This significantly impairs the patient’s quality of life and there is currently no cure, only palliative treatment options. Tissue engineering approaches are used to mimic the microenvironment of the tissue and can mediate the damaged microenvironment via bioactive compounds, to support the delivery, survival, and proliferation of new, healthy cells into the damaged environment.

**Methods:** In this study, retinyl acetate, a derivative of vitamin A, was successfully incorporated into electrospun polycaprolactone fibres.

**Results:** SEM images and characterization analyses showed that all scaffolds produced had similar characteristics, including fiber morphology and scaffold wettability. The vitamin scaffolds were shown to exert an antioxidant effect through scavenging activity of both DPPH and hydroxyl radicals *in vitro*. Critically, the antioxidant scaffolds supported the growth of human submandibular gland cells and significantly upregulated the expression of *GPx1*, an antioxidant enzyme, when cultured under both normal conditions and under a simulated oxidative stress environment.

**Discussion:** These results suggest that incorporation of retinyl acetate into electrospun fibres has may mediate the damaged microenvironment post cancer radiation therapy.

## 1 Introduction

Despite the overall decrease in cancer mortality rates, there was over 19 million new cases of cancer diagnoses worldwide in 2020 (Sung et al., 2021). Whilst current treatment options are effective in destroying cancer cells, they also inevitably cause damage to surrounding and nearby healthy cells and tissue (Hubenak et al., 2014; Wang et al., 2018; van den Boogaard et al., 2022). Such damage alters the microenvironment, causing adverse side effects that range from short-to long-term that can reduce the patient’s quality of life. With the overall mortality rate of cancer decreasing, this means that there is a significant increase in the number of patient’s living with these impeding effects. Xerostomia (dry mouth) is one significant side effect resulting from salivary gland dysfunction caused by radiotherapy and chemotherapy to treat head and neck cancer (HNC), as well as other cancers (Wilberg et al., 2014; Jensen et al., 2019). Xerostomia has major impacts on multiple daily functions, including swallowing and digestion, oral and dental hygiene, and speech, having a detrimental effect on the person’s quality of life (Yoo et al., 2014). To date, there is no preventative measures nor permanent cure, and with the vast majority of HNC patients developing xerostomia, there is great need for more effective treatments than the palliative level care currently offered (Rocchi and Emmerson, 2020).

Following irradiation, it has been established in multiple tissues, including salivary glands, that there is an almost immediate increase in the levels of cellular ROS, therefore establishing their involvement in salivary gland (or any other tissue) injury (Jasmer et al., 2020; Liu et al., 2021). The levels of ROS within the body are typically maintained by antioxidants; however, the ability of endogenous antioxidants to maintain healthy levels of ROS reduces with factors such as age (Liu et al., 2018). Increased levels of ROS leads to oxidative stress, a phenomenon that causes DNA damage and interferes with cellular processes such as proliferation and cell signaling pathways, as well as reducing the body’s natural antioxidant effect on cancer cells (Nourazarian et al., 2014). The effect of antioxidants on excess levels of ROS has been investigated in literature (Salganik, 2001; Poljsak et al., 2013). In general, antioxidants scavenge free radicals to prevent their involvement in oxidative reactions. These molecules are found both naturally within the body and are also ingested through diet. More recently, the antioxidant effect of vitamins and their derivatives has gained interest. Such compounds are of particular interest as they have been found to exert, amongst other beneficial effects, anticancer properties (Mamede et al., 2011). As with many delivery systems, one of the biggest hindrances to the use of antioxidants is the ability to deliver an effective dose to the target site (Ratnam et al., 2006). As such, the design of a delivery carrier is highly important in the translation of antioxidant therapies.

The use of biomaterials and tissue engineering to act as a carrier for compound delivery which shown promise in recent times in a variety of tissues, such as the heart, liver, kidney, and bone; as well as applications in cancer therapeutics (Contreras-Cáceres et al., 2019; Grant et al., 2019; Flaig et al., 2020; Zhao and Cui, 2020). Promising techniques used to achieve this include electrospinning, hydrogels, freeze drying, and 3D printing (Chen et al., 2012; Ding et al., 2019; Unnikrishnan et al., 2021). In particular, electrospinning to produce fibrous polymer scaffolds has been greatly studied due to their favorable properties, especially the ability to tune the properties and characteristics of the scaffold. Features such as fiber alignment, diameter, porosity, and topography can be controlled, all which influence the performance of the scaffold (Denchai et al., 2018). Additionally, small molecules such as drugs and growth factors, as well as tissue extracellular matrix (ECM) can be incorporated into the fibres (Ranganath and Wang, 2008; Wang et al., 2016; Bhattarai et al., 2019; Reid and Callanan, 2019). Carriers such as polymer fibres offer a promising alternative route to directly deliver not only drug compounds, but also volatile molecules such as vitamins, to a target site where they can repair and regenerate damaged tissue, with the potential of restoring function. Additionally, incorporating these compounds into fibres can protect them from degradation, allowing delivery not only in a controlled manner, but also tailored to the application of the scaffold (Yi et al., 2016; Mele, 2020; Vilchez et al., 2020; Musiał-Kulik et al., 2021).

In this study, electrospun polycaprolactone (PCL) scaffolds with different retinyl acetate (RA) concentrations were fabricated and their functional properties characterized. *In vitro* testing with human submandibular gland (HSG) cells, a stable and functional salivary gland cell line, under both normal and simulated oxidative stress environments was performed. This was used to determine cell viability and compatibility to ensure that the scaffolds cause no further or any new damage to the radiation damaged site.

## 2 Materials and methods

### 2.1 Scaffold production

PCL (Mn = 80,000 Da) (Sigma) was dissolved in hexafluoroisopropanol (HFIP) (Manchester Organics) with different amounts of retinyl acetate (RA) (Sigma) in a glass vial, protected from light, to give 0%, 0.1% and 0.5% RA, 10% PCL (w/v) in HFIP polymer solution. Each solution was left on a tube roller overnight at room temperature to create a homogenous solution. To prepare each scaffold, the appropriate polymer solution was loaded into a 20 mL syringe attached to a syringe pump and electrospun using an IME technologies EC-DIG electrospinning system. A 0.3 mm inner diameter brass needle was used at a 15 cm distance from an aluminum foil covered rotating mandrel (8 cm diameter, 250 RPM), which was used to collect the fibres. A total volume of 4.0 mL polymer solution was spun at a rate of 0.8 mL/h across a distance of 100 mm. The scaffold mat was left to air-dry in a fume hood overnight to allow any residual solvent to evaporate.

### 2.2 Scaffold characterization

#### 2.2.1 Scaffold morphology

The morphology and diameters of the electrospun fibres was characterized using scanning electron microscopy (SEM) analysis. 10 mm scaffolds (*n* = 3) were punched and gold sputter coated for 30 s (Emscope SC500A) prior to imaging. SEM images were obtained using 15 kV BSE accelerating voltage (Hitachi TM4000Plus SEM). Average fiber diameter size was measured using ImageJ software (150 fiber diameters for each sample).

#### 2.2.2 Mechanical testing

Tensile testing of the scaffolds was performed using an Instron material testing machine (Instron Model 3,367, 50 N load cell) with Bluehill 3 software. Scaffolds were cut into strips (80 mm × 10 mm) and mounted 20 mm at each end to give a gauge length of 40 mm. The samples (*n* = 5) were then stretched at a rate of 50% strain per minute until failure. Scaffold thickness was measured using handheld digital vernier calipers. The incremental Young’s Modulus was calculated for each sample in 5% increments to a total of 20% strain.

#### 2.2.3 Water contact angle

A 5 μL water droplet was pipetted onto the top of each scaffold (*n* = 5). Images were taken every 0.2 s using a DMK 41AU02 (ImagingSource) camera at 5 Hz. The contact angle was then measured using the ImageJ Contact Angle plugin.

### 2.3 Antioxidant assays

#### 2.3.1 DPPH assay

The antioxidant activity of unseeded scaffolds was determined using 2,2-diphenyl-1-picrylhydrazyl (DPPH) (Sigma). 10 mm circular samples were incubated in EtOH (70%, 10 min) (Sigma) followed by PBS (10 min, x3) (Gibco) to sterilize. 100 μM DPPH solution in methanol (ThermoFisher Scientific) was prepared and protected from light. 1.0 mL DPPH solution was added to each sample (*n* = 5). Plates were protected from light and incubated at room temperature (RT) for 30 min. The absorbance of the samples was measured using a Clariostar^®^ Plus microplate reader (BMG LABTECH) at 517 nm. The percentage of DPPH scavenging was calculated using Eq. 1, where *A*_*B*_ and *A*_*S*_ are the absorbances of the blank and sample, respectively.

$$\%\;DPPH\;Scavenging=\left(\frac{{A_{B}-A}_{S}}{A_{B}}\right)\times 100$$ (1)

Equation 1 Percentage of DPPH Scavenging Activity.

#### 2.3.2 Hydrogen peroxide assay

100 and 25 μM hydrogen peroxide solutions were prepared by adding 0.3 μL hydrogen peroxide (9.8 M) (Sigma) to 30 mL MEM (non-supplemented) to prepare the 100 μM solution, and then 5 mL of this was added to 20 mL MEM non-supplemented media (Gibco). EtOH (70%) was used to lift the scaffolds from the foil before they were added to the appropriate wells. 10 mm scaffolds were sterilized in 0.5 mL EtOH (70%) for 10 min. Spent solution was aspirated to waste and scaffolds were subsequently rinsed with 1 mL PBS for 10 min in triplicate. 1 mL of the appropriate media (supplemented MEM, 25 μM hydrogen peroxide, 100 μM hydrogen peroxide) was added to each well and the plates incubated at 37°C, 5% CO_2_ for the appropriate duration. 1 mL samples of the three different medias were taken for the Day 0 samples and frozen at −80°C. At the 24 h, day 3 and day 7 timepoints, 70 μL samples were collected and frozen.

The hydrogen peroxide assay was performed using a fluorescent hydrogen peroxide assay kit (Sigma-Aldrich), following the manufacturers protocol. Briefly, samples were incubated with red peroxidase and horseradish peroxidase in assay buffer for 30 min at RT, protected from light. The fluorescence intensity of the samples was then measured using a Clariostar^®^ Plus microplate reader (BMG LABTECH) at *λ*_ex_ = 540 nm and *λ*_em_ = 590 nm.

### 2.4 *In Vitro* cell analysis study

#### 2.4.1 Scaffold sterilization and HSG cell seeding

10 mm scaffolds were punched and EtOH (70%) was used to lift the scaffolds from the foil backing before the scaffold mats were transferred to 48 well plates. Scaffolds were then incubated in 1 mL EtOH (70%) at RT for 30 min and rinsed with PBS (10 min) in triplicate. Scaffolds were then incubated in 0.5 mL minimum essential media (MEM) cell culture media (37°C, 5% CO_2_) for 1 h. Following this incubation period, scaffolds were left in the biohood to air dry for a minimum of 30 min.

Human Submandibular Gland (HSG) cells (HeLa derivative) were obtained from the American Type Culture Collection (ATCC) via Public Health England. The cells (passage 7) were cultured to 80% confluence in a T-75 flask with MEM supplemented with 10% (v/v) new-born calf serum (NBCS), 1% (v/v) L-glutamine and 1% penicillin-streptomycin (All Gibco with the exception of NBCS (Sigma)). Cells were seeded at a density of 40,000 cells per scaffold (50 μL cell bolus added to each scaffold), as well as on glass slides and tissue culture plastic (TCP) as controls and incubated for 1 h. Wells were topped up with 550 μL media and glass slides covered with 20 mL media and incubated (37°C, 5% CO_2)_. After 24 h, media was replenished with either 500 μL of either normal supplemented media or H_2_O_2_ supplemented media (100 μM, prepared as per Section 2.3.2). This change in media was noted as day 0 for the purpose of the cell study. An additional 500 μL media was added to all remaining samples using normal media on day 3.

#### 2.4.2 Cell viability

Cell viability was determined using CellTitre-Blue^®^ assay (Promega), conducted as per the manufacturer’s instructions at 24 h, Day 3 and Day 7. In short, cell seeded scaffolds (*N* = 5) were transferred to a fresh suspension plate with 400 µL fresh media and 100 µL CellTitre-Blue and incubated at 37°C, 5% CO_2_ for a total of 3 h. 100 μL of each sample was transferred to a 96 well plate and fluorescence measured at 560/590 nm using a Clariostar^®^ Plus microplate reader (BMG LABTECH).

#### 2.4.3 DNA quantitation

Seeded scaffolds were dissolved via papain digest in a solution containing papain (2.5 U/mL), cysteine (5 mM) and EDTA (5 mM) (all Sigma) at 24 h, Day 3 and Day 7. Scaffolds were incubated in 0.5 mL solution at 60°C and left overnight to fully dissolve. Samples (*N* = 5) were then used in the Quant-iT™ Picogreen^®^ assay (Thermo-Scientific), as per the manufacturer’s instructions, to determine the amount of dsDNA present in each scaffold. The fluorescence of the samples was read at 490 nm (*λ*_ex_ 480 nm, *λ*_em_ 520 nm) using a Clariostar^®^ Plus microplate reader (BMG LABTECH).

#### 2.4.4 Live/dead staining

Each sample was rinsed in triplicate with PBS before being fixed with formalin (10%, 500 μL) (Sigma) in the incubator (37°C, 5% CO_2_) for 30 min. Following incubation, scaffolds were rinsed twice with PBS (10 min). The stain was prepared by creating working stocks of both dyes. 0.5 μL calcein (Invitrogen) was added to 1 mL PBS; and 1.0 μL ethidium (EthD) (Invitrogen) was added to 1 mL PBS and protected from light. 20 μL EthD and 5 μL Calcein working solutions were added to 10 mL PBS. 150 μL of stain was added to each scaffold and incubated at RT, protected from light, for 30 min. Following incubation, scaffolds were rinsed twice with PBS (10 min). Images were taken using a Zeiss AxxioImager epifluorescence microscope at ×25 magnification. (*N* = 5).

#### 2.4.5 DAPI/phalloidin staining

Samples were incubated in 0.2% Triton X-100 (Sigma) at RT for 10 min. 1% v/v Phalloidin conjugate in PBS with 0.5% bovine serum albumin (BSA) was prepared by combining 1% BSA (Sigma) in 20 mL PBS and 0.2 μL Phalloidin 514 (Sigma). 100 μL was added to each scaffold and incubated at RT, protected from light, for 1 h. Phalloidin solution was aspirated to waste and samples were rinsed in triplicate with PBS (10 min). 4’6-diamino-2-phenylindole (DAPI) solution was prepared by adding 20 μL DAPI stock (Thermofisher) to 20 mL PBS. 100 μL was added to each scaffold and incubated at RT, protected from light, for 10 min. Following the incubation period, scaffolds were rinsed twice with PBS (10 min) before images were taken using a Zeiss AxxioImager epifluorescence microscope at ×40 magnification. Images were then processed using ICY software (*n* = 5).

### 2.5 Gene expression

RNA was extracted from the samples using Tri-Reagent (Invitrogen) and purified using an RNA extraction kit (Promega). Gene expression was determined using reverse transcriptase polymerase chain reaction (RT-PCR).

Forward and reverse primer sequences were designed using PromoBlast and custom made by Merck (Table 1). Expression of aquaporin-5 (AQP5), B-cell lymphoma-2 (BCL-2), and glutathione peroxidase 1 (GPx-1) in the HSG cells grown in both normal and hydrogen peroxide medias was measured. The levels of gene expression were measured using the 2^−ΔΔCT^ method and are given relative to the reference gene glyceraldehyde 3-phosphate dehydrogenase (GAPDH) at D3 in the respective media type.

**TABLE 1:** Forward and reverse primer sequences used in PCR.

Gene	Primer	Sequence (5′-3′)	References
Aquaporin-5	*AQP5* (forward)	GCT​CAC​TGG​GTT​TTC​TGG​GTA	Yan et al. (2017)
	*AQP5* (reverse)	CCT​CGT​CAG​GCT​CAT​ACG​TG	
Glutathione peroxidase 1	*GPx1* (forward)	CGC​CAA​GAA​CGA​AGA​GAT​TC	Jaiboonma et al. (2020)
	*GPx1* (reverse)	CAA​CAT​CGT​TGA​GAC​ACA​C	
B-cell lymphoma-2	*BCL2* (forward)	ATC​GCC​CTG​TGG​ATG​ACT​GAG​T	Jiang et al. (2020)
	*BCL2* (reverse)	GCC​AGG​AGA​AAT​CAA​ACA​GAG​GC	
Glyceraldehyde 3-phosphate dehydrogenase	*GADPH* (forward)	GTC​TCC​TCT​GAC​TTC​AAC​AG	Reid et al. (2020)
	*GADPH* (reverse)	GTT​GTC​ATA​CCA​GGA​AAT​GAG	

### 2.6 Statistical analysis

All data was subjected to statistical analysis conducted by one-way ANOVA with Tukey’s *post hoc* test (OriginLab). Statistical differences considered were as follows: *p < 0.05, **p < 0.01, and ***p < 0.001. Data is reported as mean ± standard deviation (SD). Error bars in figures represent the SD.

## 3 Results

### 3.1 Scaffold fabrication and characterization

**FIGURE 1:**
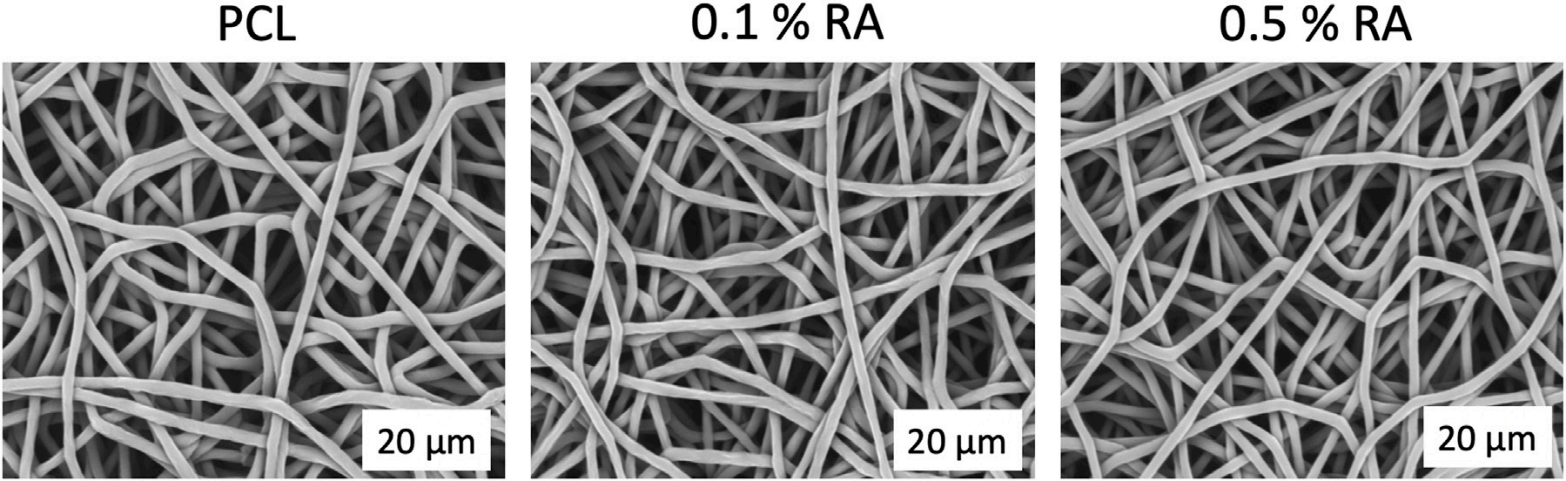
SEM images of each scaffold group taken at ×4,000 magnification.

RA was successfully incorporated into electrospun PCL polymer fibres. SEM image analysis confirmed that the fibres obtained all had similar morphology (Figure 1). The fibres were smooth and randomly aligned. A summary of characteristics for each scaffold is shown in Table 2. Minimal change was observed between the groups, indicating that the inclusion of RA in the PCL fibres did not have any deleterious effect on the scaffold morphology.

**TABLE 2:** Scaffold Properties for each scaffold group.

Scaffold	Water contact angle (°)	Fiber diameter (μm)	Ultimate tensile strength (MPa)
PCL	113.91 ± 0.28	1.71 ± 0.11	2.59 ± 0.14
0.1% RA	113.55 ± 0.28	1.67 ± 0.14	2.62 ± 0.24
0.5% RA	113.50 ± 0.77	1.60 ± 0.11	2.17 ± 0.15

There was significant difference at the *p* < 0.01 level between the fiber diameters of each group (*n* = 150) and *p* < 0.001 between both PCL and 0.1% RA with the 0.5% RA scaffold; with diameters ranging from 1.60 to 1.71 µm. Compared to the PCL fiber, there was only a 2.3% and 6.4% reduction in fiber diameter size for the 0.1% RA and 0.5% RA scaffolds, respectively.

Water contact angle (WCA) confirmed the hydrophobic properties of the scaffold. PCL is known to be hydrophobic (Deng et al., 2019). WCA measurements were taken to determine if the inclusion of RA into the PCL fibres had any impact at all on the hydrophobicity of the fibres, as this could potentially impact cell adherence when culturing the cells on the scaffold, which would therefore influence the rest of the study. No significant difference was found between the WCA of the three groups.

When comparing the ultimate tensile strength (UTS) of the scaffolds, there was statistical significance of *p* < 0.01 between the two vitamin-containing groups and statistical significance at the *p* < 0.05 level between the PCL and 0.5% RA scaffolds.

**FIGURE 2:**
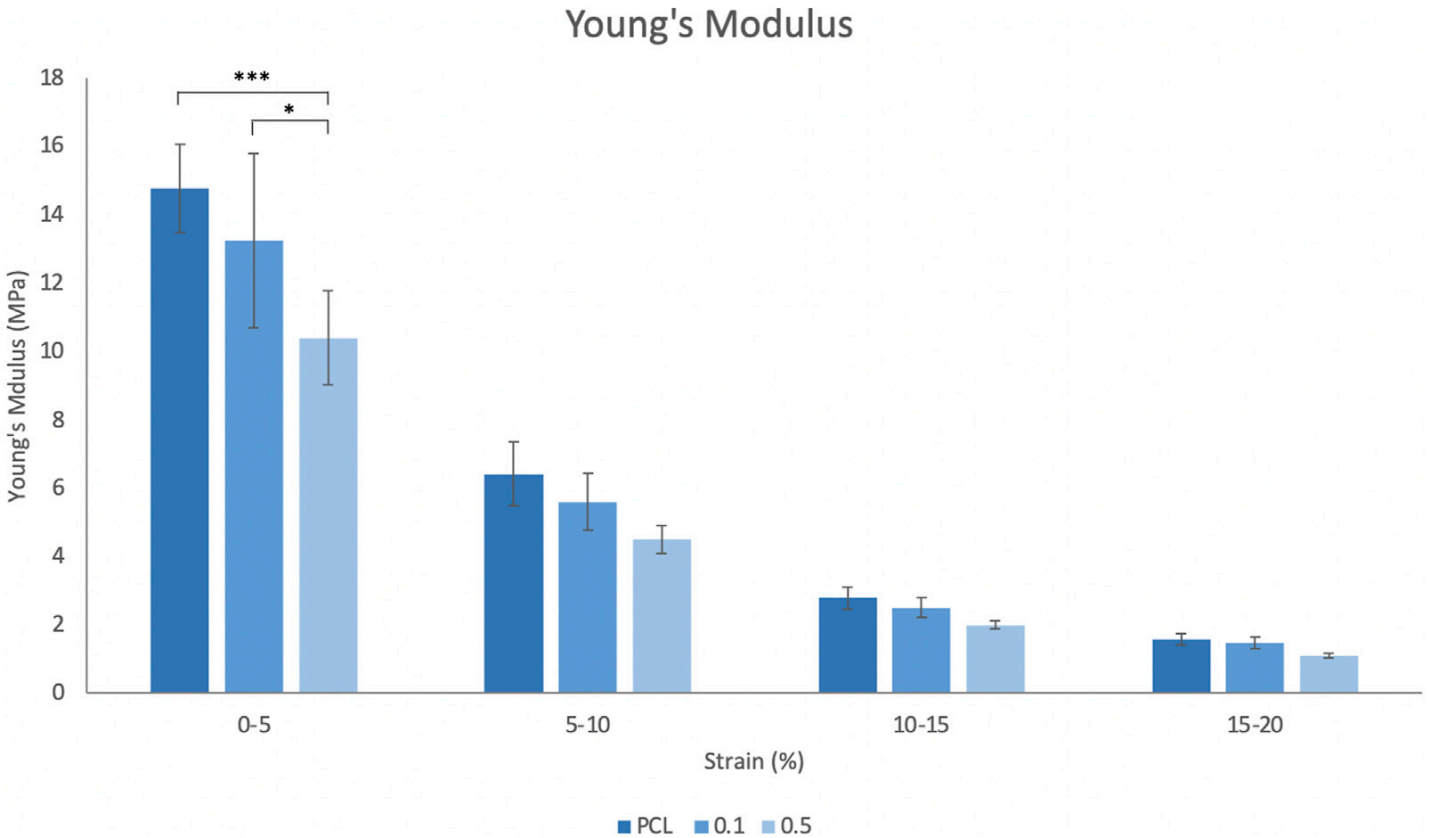
Incremental Young’s modulus measured from 0% to 20% strain. Error bars represent the SD.

Figure 2 displays the Young’s modulus (YM) for each scaffold group, measured over four increasing 5% strain increments. As shown at 0%–5% strain, the incorporation of RA in the fibres led to a decrease in YM, with the PCL only scaffolds being the stiffest at each range. This trend was observed in all strain increments up to 20% and overall, the material stiffness statistically reduced as the strain applied increased.

### 3.2 Antioxidant activity

**FIGURE 3:**
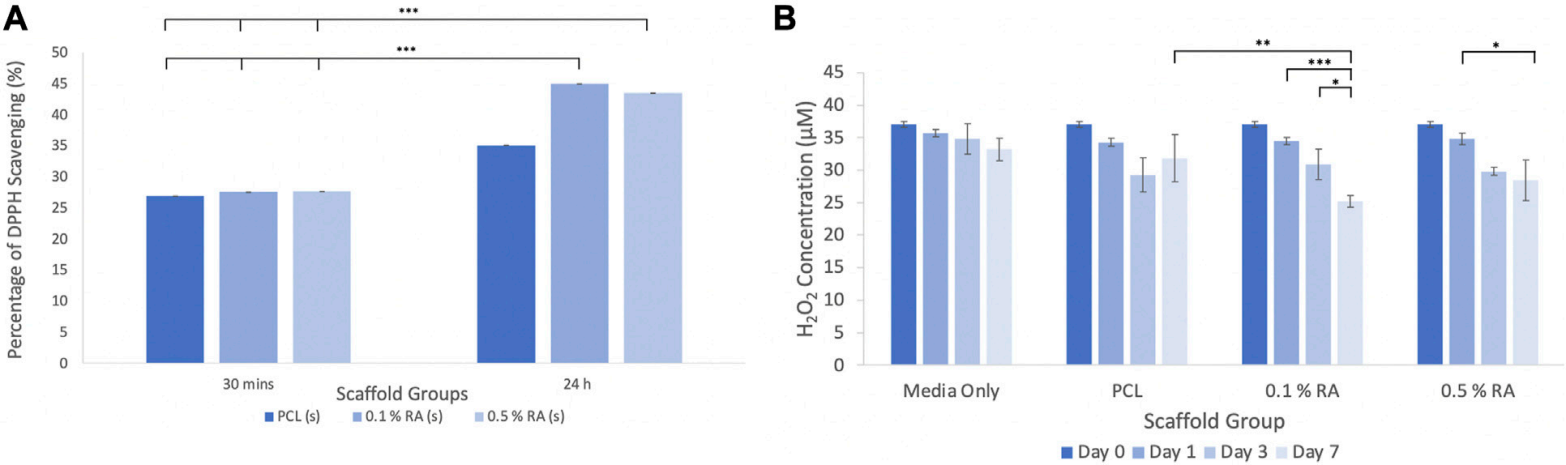
**(A)** Results of the DPPH scavenging assay **(B)** results of the fluorometric H_2_O_2_ assay. Error bars represent the SD.

Vitamin A and its derivatives, such as RA, are known to exert an antioxidant effect (Wu et al., 2017; Celebioglu and Uyar, 2020; Khadim and Al-Fartusie, 2021). DPPH and H_2_O_2_ assays were used to establish that the RA scaffolds were capable of producing an antioxidant effect (Figure 3). Whilst the DPPH assay is typically used for natural compounds, researchers have more recently employed its use for determining the antioxidant activity of vitamins, such as retinyl acetate (Celebioglu and Uyar, 2020).

#### 3.2.1 DPPH assay

The DPPH assay (Figure 3A) showed that at 30 min, both the PCL control and the RA containing scaffolds showed similar absorption, with approximately 27% scavenging activity. The scavenging activity was slightly higher in the RA scaffolds, albeit marginally, however this was not statistically significant. As there was little difference between the groups, the scaffolds were incubated again for a total period of 24 h and the analysis performed again. After 24 h, scavenging activity increased to between 35% and 45% and there was a more notable difference between the RA scaffolds and the PCL control.

#### 3.2.2 Hydrogen peroxide assay

Over the duration of the test, the concentration of hydrogen peroxide was reduced when the RA containing scaffolds were incubated in H_2_O_2_ media (Figure 3B). It can be seen that there was a significant reduction between each timepoint taken from the 0.1% and 0.5% RA scaffold samples. In particular, the 0.1% RA scaffold was shown to be the most effective in reducing the level of H_2_O_2_. This result aligns with the DPPH assay to show that the RA scaffolds are capable of producing an antioxidant effect.

### 3.3 *In Vitro* analysis

**FIGURE 4:**
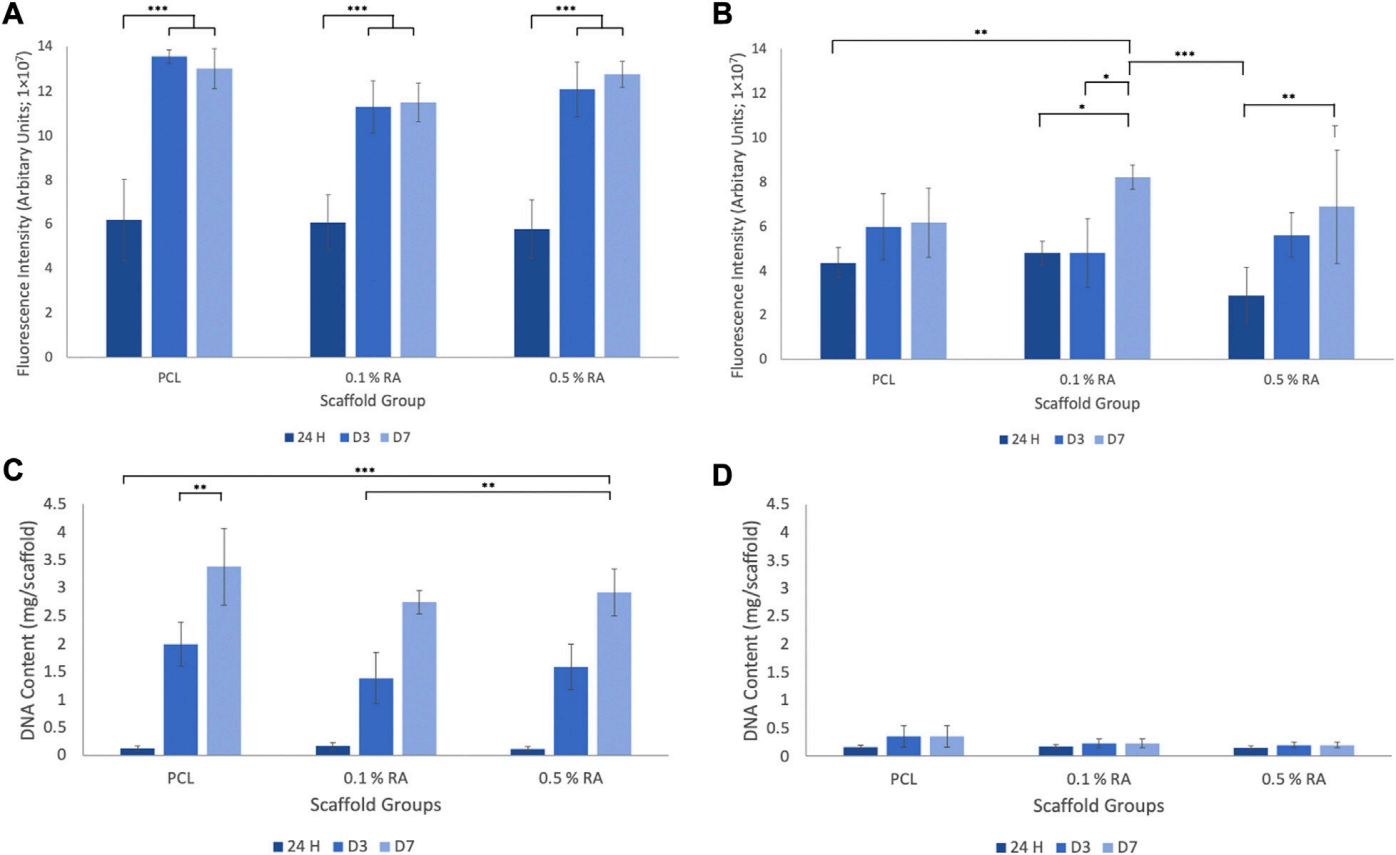
**(A)** Cell viability in normal media **(B)** cell viability in H_2_O_2_ media **(C)** DNA quantitation in normal media **(D)** DNA quantitation in H_2_O_2_ media. Error bars represent the SD.

A series of assays were performed on cells grown on each of the scaffolds to confirm that RA and the scaffolds themselves were not cytotoxic to the cells. As shown in Figures 4A, B, cell viability in both normal and H_2_O_2_-doped media increased over the duration of the study.

In the cell viability assay for cells cultured on the scaffolds in normal media (Figure 4A), there was statistical significance between 24 h and days 3 and 7 in all groups. The results clearly show that cells were able to survive on each scaffold group and the incorporation of RA into the fibres did not have a deleterious effect. TCP results are not shown, but showed that the H_2_O_2_ media did, as expected, decrease the viability of the cells initially, however the cells were shown to recover. This can be found in the Supplementary Material (SF1). The correlating DNA quantitation results (Figure 4C) confirms that cells were successfully proliferating throughout the duration of the study in all scaffold groups.

The incorporation of H_2_O_2_ into the incubation media was used to successfully induce an *in vitro* ROS environment. This was key in ensuring that the scaffolds were still capable of supporting the proliferation of cells within a damaged environment, similar to what would be seen post-irradiation. This showed a decline in viability. When incubated in H_2_O_2_ media, seeded scaffolds were still viable, as shown in Figure 4B, however the DNA content (Figure 4D) was significantly lower, and there was no statistical significance seen between any of the groups at any timepoint.

### 3.4 Cell imaging

**FIGURE 5:**
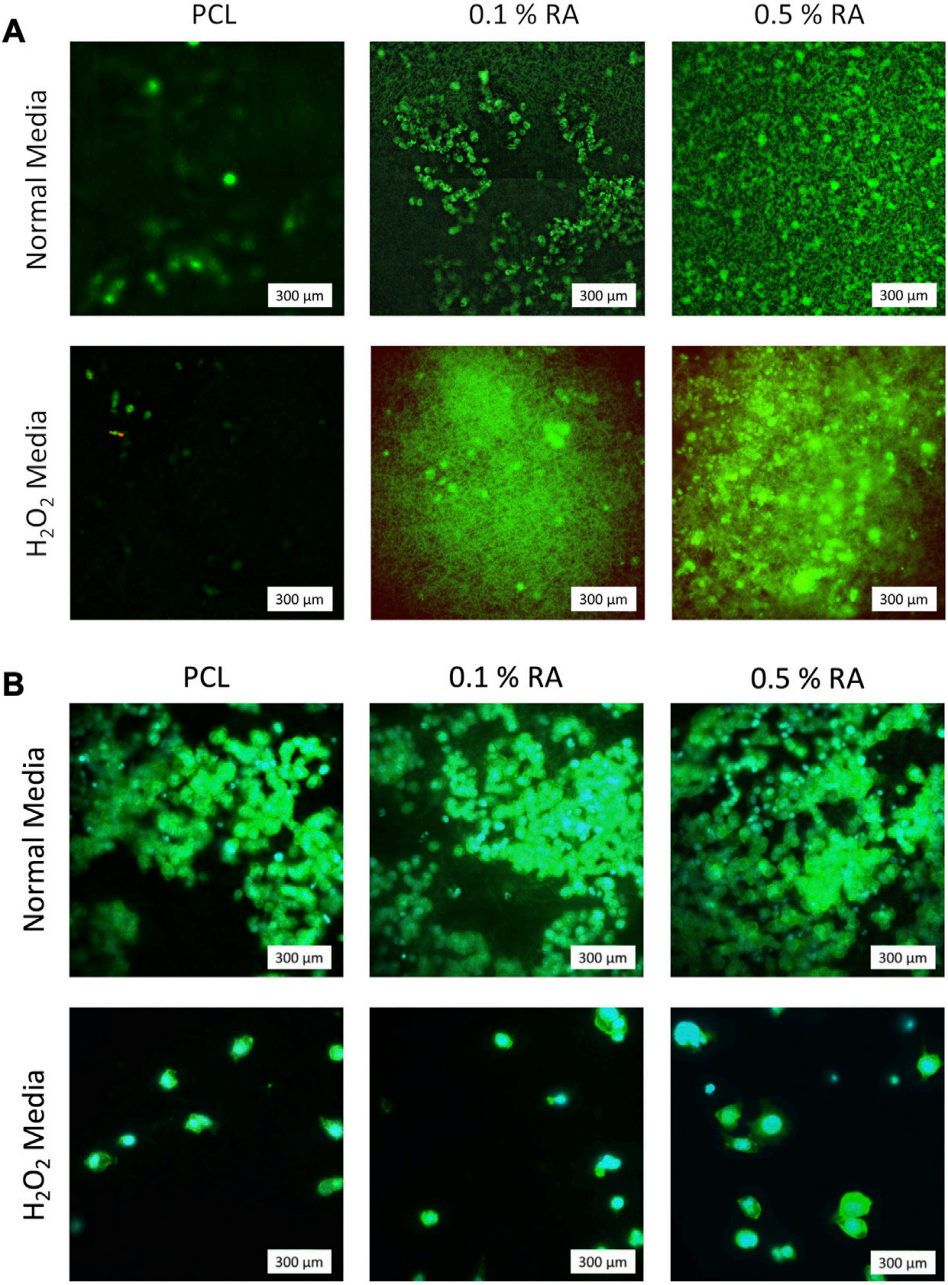
Representative images of **(A)** Live/Dead staining at D7. Green represents live cells; red represents dead cells **(B)** DAPI/Phalloidin staining at D7. Cyan represents nuclei (DAPI); green represents actin filaments (Phal.).

Both Live/Dead and DAPI/Phalloidin staining (Figures 5A, B, respectively) confirm that the cells were adhered to and successfully proliferated on the surface of the scaffold. In the Live/Dead staining of seeded scaffolds incubated in H_2_O_2_ media (Figure 5A), it can clearly be seen that whilst cells are still able to proliferate in the H_2_O_2_ media, it inhibits the rate at which it does so. There are two dead cells that were observed in the seeded PCL scaffold incubated in H_2_O_2_ sample, showing that the scaffolds are not toxic to the HSG cells. There were no dead cells observed on the seeded scaffolds containing RA, indicating that the inclusion of the vitamin had no significant detrimental effect.

### 3.5 Gene expression

**FIGURE 6:**
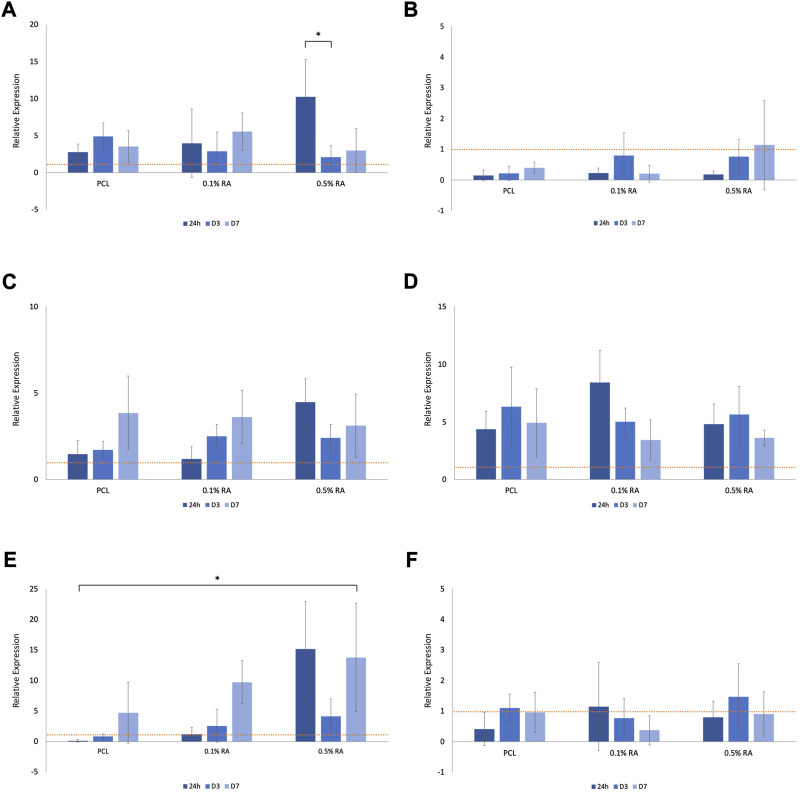
Relative expression of **(A)** *AQP5* in normal media **(B)** *AQP5* in H_2_O_2_ media **(C)** *BCL2* in normal media **(D)** *BCL2* in H_2_O_2_ media **(E)** *GPx1* in normal media **(F)** *GPx1* in H_2_O_2_ media, normalized to D3 TCP (represented by the horizontal dashed line in each graph). Error bars represent the SD.

Expression of genes associated with salivary glands and their function, as well as one for antioxidant activity, were determined using RT-PCR assays (Figure 6). Results were normalized to tissue culture plastic (TCP) at D3 for each gene. This is represented by the horizontal dashed line in the PCR graphs.

*AQP5* is a water channel, expressed by the secreting acinar cells and is an essential component of functional salivary gland epithelium; it is expression is routinely used to assess salivary gland function and regeneration (Matsuzaki et al., 2012; Emmerson et al., 2018). *AQP5* expression was notably reduced in the cells grown in hydrogen peroxide media (Figure 6B), in comparison to those grown in normal media (Figure 6A). Statistical significance (*p* ≤ 0.001) was found between the 0.5% RA scaffolds in the two different media types at 24 h.

Expression of the apoptosis marker *BCL2* increased in the cells grown in hydrogen peroxide (Figure 6D), when compared to cells grown in normal media (Figure 6C). There was no significant difference seen within groups, nor between timepoints. At 24 h statistical difference (*p* ≤ 0.001) was seen between 0.1% RA scaffolds in normal and H_2_O_2_ media. Interestingly, while expression of *BCL2* dropped with time in the 0.1% RA scaffolds, in the higher RA concentration, expression increased.

Across all groups, expression of *GPx1*, an antioxidant marker, was highest at D7 (Figure 6E), increasing as the amount of antioxidant in the scaffold increased. There was still some expression, particularly at D3, in the hydrogen peroxide media, with PCL and 0.1% RA being upregulated in comparison to the same groups in the normal media (Figure 6F). Statistical difference at the *p* ≤ 0.001 level was observed between normal and H_2_O_2_ conditions with both the 0.1% and 0.5% RA scaffolds at 24 h, and the 0.5% RA scaffolds on D7. For the 0.1% RA scaffolds at D7, the statistics showed that *p* ≤ 0.05.

## 4 Discussion

Herein, we have investigated the incorporation of RA into PCL, a common (FDA approved) biomaterial used for tissue engineering, fibres to produce scaffold mats capable of producing an antioxidant effect. RA is a derivative of vitamin A, which has not only has been shown to exert antioxidant and anticancer properties, but also has been found to be a significant signaling molecule in the process of morphogenesis in mammalian submandibular salivary glands, through direct action in the tissue itself (Wright et al., 2015; Abashev et al., 2017). To the best of our knowledge, this is the first application of an electrospun antioxidant scaffold designed to regulate ROS levels in the salivary gland with the potential to support survival and proliferation of SG cells.

Significantly, the DPPH and hydrogen peroxide fluorometric assays produced results which confirmed that the scaffolds produced were capable of exerting an antioxidant effect. Previously published literature has shown that the scavenging activity of tissue engineered scaffolds can be increased by incorporating antioxidant compounds. Kheradvar et al. fabricated nanofibers containing vitamin E (VE) to be used for promoting repair and wound healing (Kheradvar et al., 2018). They found that increasing VE concentration lead to an approximate 48% difference in antioxidant activity between 1% and 5% VE within the first 4 h. A similar trend was observed in our study where results showed an 18% increase between 0.1% and 0.5% RA, however this was within the first 24 h. Interestingly, there is a three-fold difference between these sets of results, with a six-fold increase in duration. Naturally, there are several factors that can be attributed to these differences. Firstly, PCL is insoluble in methanol (used to prepare the DPPH solution in the assay), whereas poly (vinyl alcohol) (PVA) is readily soluble. This means that PVA fibres from the Kheradvar *et al.* system will degrade more rapidly, releasing and exposing more vitamin from within the fibres to the DPPH. The fiber sizes and morphology will also influence their function, as will the differences between the two vitamins.

Incorporation of small molecules and compounds into polymer scaffolds overcomes the hindrance faced in current delivery methods with regards to absorption, distribution, metabolism, and excretion (ADME) properties and bioavailability/biocompatibility, which inhibit the delivery of a compound to its target site. However, one caveat of vitamins, particularly vitamin A, is their volatility. Vitamin A and its derivatives, including retinoic acid and RA, are sensitive to light, temperature, humidity, and oxygen (Kim et al., 2008). It has been shown previously that incorporation of such molecules into electrospun fibres using a blend polymer solution provides some protection against these factors (Adeli-Sardou et al., 2019). Small molecules are typically eluted from scaffolds in an initial burst release, usually over the initial few days, followed by a prolonged release period that can last anywhere in the range of days to months, as reported in the literature (Lee et al., 2012; Yao et al., 2014; Adeli-Sardou et al., 2019).

In this study, minor differences were observed in the fiber diameter between the groups in this study (*p* < 0.05). These differences in fiber diameter, despite utilizing the same fabrication process, can be attributed to several conjunctive parameters such as environmental conditions such as temperature and humidity. Such parameters, as well as others including solvent choice, percentage (w/v) polymer and electrospinning parameters, can be altered (individually or collectively), in order to design specific scaffolds with characteristics tailored to suit the application of the scaffold. The sensitivity of the fabrication method leading to such deviations can subsequently make reproducibility extremely difficult, with even a slight alteration in any of the parameters affecting the fibres produced. However, in this study, there was a difference of only ∼6% between the smallest and largest fiber diameters, implying good reproducibility. Published literature has studied the effects of fiber diameter on cell viability and gene expression, but these differences are usually seen at much greater differences in fiber diameter (Burton et al., 2018; Bate et al., 2020; Reid et al., 2020). No degradation study of the fibres was performed within this study. The degradation of PCL scaffolds has been thoroughly investigated throughout the years, with numerous long-term degradation studies performed, all of which show minimal degradation of PCL over an extended period of time. For example, in a study by Lam et al., there was a maximum degradation of 7% over 6 months *in vivo*, with no visual differences in scaffold morphologies (Lam et al., 2008). Additionally, the degradation of PCL is deemed safe enough for the polymer to be FDA approved for biomedical *in vivo* applications (Woodruff and Hutmacher, 2010).

Similarities in mechanical properties of the material and tissue are considered an important parameter when designing a material for implantation. However, as there is minimal strain exerted within the salivary gland (SG) as it functions, the tensile properties of a scaffold for such applications are not as significant a consideration as it would be for other tissues, as the stiffness of the material is not going to impact the actual function of the SG tissue as it does in, for example, the beating heart or in load-bearing bone. In this study, the slight differences observed between the YM of each group is attributed to the incorporation of RA into the fibres, as the amount of PCL used in each group is consistent between all groups at 10% w/v. In order to consider incorporation of a graft like this, should it be used in a clinical application post-cancer treatment, it must have enough physical strength and good handling ability to be used in a surgical approach. The strength maintained within the scaffolds (all groups) is an acceptable level in terms of handling and being able to use and shows comparable results to similar studies in terms of the UTS of pure PCL and the reduction seen with the incorporation of an additional compound (Yi et al., 2016; Arbade et al., 2019). There was no major mechanical integrity loss within the system. The stiffness of the scaffolds is quite high in terms of physiological relative stiffness to the healthy gland itself, which typically has a Young’s modulus value in the range of 15–20 kPA (Iyer et al., 2021). While this is a major difference, the purpose of the scaffold is not to match nor mimic the healthy environment, but to deliver a product, in this case retinyl acetate. Furthermore, the salivary gland often becomes fibrotic in the weeks and months after radiotherapy, which results in a stiffer cellular environment, which will likely be more similar to such a scaffold (Ying et al., 2007; Jasmer et al., 2020).

It has been well established that PCL fibres are hydrophobic, and this was seen in the WCA of the scaffolds produced here. However, whilst the WCA of all groups was approximately 113°, in other studies the WCA has been seen to drop when components have been added to the scaffolds. In a study by Arbade *et al.*, incorporation of Emblica officinal decreased the hydrophobicity of the scaffolds; however, the RA scaffolds fabricated in this study had improved wettability (Arbade et al., 2019). The degree of wettability can cause issues with cell adhesion and subsequently cell metabolism (Rosales-Leal et al., 2010; Arbade et al., 2019). However, in this study the HSG cells were able to adhere to the scaffolds. If this had been an issue, plasma coating could have been performed to increase the hydrophilicity of the scaffolds (Siri et al., 2010).

Whilst the DAPI/Phalloidin staining indicates that there is a reduction in cell numbers when exposed to H_2_O_2_ this is not observed in the Live/Dead images. Based on the arbitrary Live/Dead imaging (Figure 5A), it seems that RA reacts with calcein (green) in the stain. Upon closer inspection of the images with the antioxidant scaffolds, what initially appears to be background noise actually shows that the calcein from the live/dead stain has adhered to the scaffold fibres. The visibility of the fibres stained with calcein increases with increasing concentration of RA, implying that a reaction is occurring between the RA on the surface of the fibres with the dye. The green fibres cannot be seen in the PCL control images, confirming that the fluorescence is due to the retinyl acetate. To the best of our knowledge, this finding has not been stated in any previous literature. Whilst these images are representative and live cells are still observed on the scaffold as expected, this apparent reaction between the RA and calcein should be taken into consideration and taken as an artefact of using calcein as a live stain in the presence of RA.

Gene analysis was performed on a range of genes found within the SG (Figure 6). Genes were selected based on function that was most applicable to the function of the salivary gland and that would reflect the impact that the harsh, ROS-mimicking environment has on the growth of the HSG cells. *AQP5*, *BCL2*, and *GPx1* genes code for proteins associated with water-channels, apoptosis, and antioxidant activity, respectively. RT-PCR was performed to determine gene expression of cells grown in both normal media and H_2_O_2_-doped media and normalized to tissue culture plastic (TCP) at D3 for each condition.

As expected, the expression of *AQP5* was substantially reduced in the oxidative stress simulated environment. As *AQP5* is a water channel protein essential in the production of saliva it would be expected that expression, and subsequently function, of this protein is reduced in the ROS-mimicking environment (Satoh et al., 2013). This relates to the reduction in salivary gland function observed following irradiation treatment (Kojima et al., 2011). Despite this, the gene is still present, and a general upwards trend was observed with increasing RA concentration, indicating RA can preserve the functionality of the SG. Maintenance of genes and their functions within cells is important for many reasons, such as specific molecule and protein production for healthy cell development, as well as predisposition towards certain diseases or illnesses. As a water channel protein, any downregulation of *AQP5* would worsen the symptoms of xerostomia experienced by the patient (Li et al., 2006; Wei et al., 2022).

*BCL2* promotes cell survival and increased expression is associated with an increased resistance to apoptosis (Noy, 2010; Marie Hardwick and Soane, 2013; Quintero Barceinas et al., 2015). This means that the activation of the protective gene stops the cells from programmed cell death. Within the normal media, expression generally increases from 24 h to D7, with higher expression in the RA containing scaffolds compared to the PCL control group. This suggests that RA does play a role in maintaining cell survival. Overall, there is greater expression of the gene in cells cultured in H_2_O_2_ media in comparison to those cultured in normal media. However, a common trend observed in the expression of *BCL2* in H_2_O_2_ media is that, in contrast to what was observed in the normal media, there is a drop observed in expression between D3 and D7. This could be due to the interaction between RA and the H_2_O_2_ in the media, but also could be the result of the increased efforts of cells trying to stop apoptosis, linking back to the reduction of cell viability seen in (Figures 4B, D). The upregulation of *BCL2* observed in the H_2_O_2_ media is logical in the sense that the cells are trying to protect themselves by blocking the apoptosis pathway, driving cell survival, and this is intensified by the harsh environment.

In normal conditions, oxidative stress causes upregulation of *GPx1*, an enzyme that oxidizes GSH to GSSG to scavenge ROS and maintain cellular redox (Dequanter et al., 2017; Zhao et al., 2022). However, this may not be the case for damaged environments. *GPx1* expression in normal media showed a similar increasing trend between timepoints in each group, with the highest expression seen in the 0.5% RA scaffold at each timepoint. In comparison, expression is significantly reduced in H_2_O_2_ media. At D3, both conditions see an increase for *GPx1* compared to 24 h, and again from D3 to D7, which may indicate that the RA has a positive effect on these cells, as *GPx1* drives antioxidant activity. Again, the important finding here is that the gene is maintained, and the presence of RA does not disrupt the production of the gene. Another notable observation is that expression of *GPx1* increases even though cell viability decreases; there are fewer live cells on the scaffold, however they are producing and exponentially increasing their antioxidant effect.

Whilst there was limited trends/statistical significance observed during RT-PCR, all genes were still functionally active under both conditions and at all RA concentrations (as well as in PCL controls), highlighting the positive ability of HSG cells to maintain gene expression, and thus function, of three characteristic genes of the SG. The scaffold did not have any negative impact on the genes expressed by HSG cells, and the results also suggest that the presence of RA promotes cell survival. Whist there are many other factors that can influence regulation of a gene, such as the concentration of antioxidant, the amount present on the surface of the scaffold, cell number and resilience, there is an overall general trend that suggests that the inclusion of the antioxidant in the scaffold fibres has a positive benefit on HSG cells cultured under oxidative stress conditions.

This study is not without its limitations. The scaffolds used in this study are composed of randomly aligned fibres. Whilst it is possible that directionally aligned fibres may be more suited to restoring flow of saliva in the gland, the random alignment of fibres in scaffolds is beneficial when seeding cells as the cells can become trapped within the pores of the scaffold mat. As the purpose of this study was to determine the compatibility between the scaffolds and HSG cells and purposed as a means of reintroducing healthy cells to a damaged environment, this was not a parameter that was considered crucial at this time. The morphology of the scaffolds is, however, an important consideration as effects such as surface topography and pore size can influence both the attachment and growth of cells on the scaffold (Lowery et al., 2010; Kim et al., 2016; Gao and Callanan, 2021).

Scaffolds were fabricated by electrospinning PCL polymer solution, where PCL pellets and RA were dissolved in HFIP. RA is insoluble in water therefore an alternative solvent had to be used. Whilst water is preferable in terms of safety for use *in vivo*, the solvent is allowed to evaporate and is also rinsed in ethanol followed by multiple PBS washes to ensure that the scaffold is completely sterile and void of any residual solvent. This method gives polymer fibres with the RA incorporated throughout the scaffold.

There is approximately 0.006 and 0.001 mg RA in the 0.5% and 0.1% RA scaffolds, respectively. However, there was no guarantee that the RA was evenly distributed across the fibrous sheet fabricated despite care taken to ensure that the polymer solution was homogenous and minimize this risk. For this reason, the exact concentration of RA in each scaffold was not determined. Additionally, RA is known to be a delicate compound, being sensitive to factors such as light and temperature (Tolleson et al., 2005). However, it has been shown that encapsulating the RA within the PCL will provide some protection to the RA by the PCL (Celebioglu and Uyar, 2020). While it would be interesting to measure the exact quantity of RA within the scaffolds, when it comes to the function of these scaffolds, we are mainly interested in how much of the RA is released from the fibres to produce the desired antioxidant effect. We predominantly focused on the external functionality of the scaffolds (through the DPPH assay) as, as previously mentioned, the amount of RA in the scaffold may not be representative of the amount present on the surface of and released by the fibres.

The cells used in this study are HSG epithelial cells, which are known to be contaminated with HeLa cells (Lin et al., 2018). This may influence the results and outcome in terms of testing the suitability of this process; however, HSG cells still provide a good representative model for salivary gland cells for determining the effect that an antioxidant may have on their survival and proliferation. There are no standard SG stem cell lines, however HSG cells are functioning SG cells that are robust enough to withstand the test conditions subjected to in this study, and to ensure that the scaffold does not further damage or induce new damage upon gland cells that have survived radiation treatment. In the future, alternative cells, such as primary epithelial cells, could be investigated to further prove function and provide a more representative model system.

Both antioxidant studies confirm that there is RA present in the fibres; however, further characterization and studies are required to confirm the amount of RA present within the fibres and its distribution throughout the fiber/scaffold. The distribution of the RA within the fibres will affect the results observed. Scaffold mats where the RA is on, or closer to, the surface of the fiber will produce a quicker antioxidant effect in comparison to fibres where the RA is more embedded. In the H_2_O_2_ assay, there appears to be a saturation limit around 40 μM, above which the concentration of H_2_O_2_ cannot be determined. Additionally, *in vitro* degradation and release rate studies would be beneficial in profiling the release of RA from the scaffolds. While these studies prove that the scaffolds exert an antioxidant effect, they do not highlight any changes that may occur to the intracellular ROS levels. In future studies, it would be of interest to include an assay, such as the DCFDA/H2DCFDA assay that shows ROS activity within the HSG cells themselves (Lee et al., 2020; Yang et al., 2021; Wang et al., 2023).

Whilst the cell viability assay measures the metabolic activity of the cells, DNA quantitation results confirm the proliferation of the cells. Both should be considered as metabolic activity within cells varies at different stages of the cell cycle and therefore can only be used as an indicator of viability, i.e., the cells can be alive but depending on their state will exert different metabolic activity. Overall, the combined results provide confirmation that the HSG cells are proliferating on the scaffolds and that this is not hindered by the inclusion of RA within the scaffold fibres.

There is evidence to suggest that the material needs time to activate the cells, as we see in the RT-PCR results of GPx1 after 3 days exposure to H_2_O_2_ and then again at D7 (Figures 6E, F). At this point, the upregulation of the gene suggests that there is an activation of antioxidant activity within the HSG cells themselves. It could be that these cells require pre-exposure to the material prior to being placed into the ROS-mimicking condition before producing an antioxidant effect. Introducing a pre-exposure time would allow an initiation period for the activation of the gene, which would be more beneficial when employed as a therapeutic approach. The positive results seen within this study provide motivation to investigate this further in future studies.

As previously mentioned, vitamin A (and its derivatives) have been shown to produce an anticancer effect (Blumenthal et al., 2000; Doldo et al., 2015). An important study in the future would entail determining if the RA scaffolds were capable of producing such an effect. This could be done *in vitro* using organoids/spheroids. A dual-action scaffold that is able to not only reduce the amount of ROS within the damaged environment but also provide an anticancer effect would be extremely beneficial.

The results outlined within show that there is promise for the use of these RA-PCL electrospun scaffolds. Future work should include testing the scaffolds with other cell types—including healthy cells and primary cells which, if successful, will provide evidence in support of an *in vivo* study. Mouse models are extensively used in the salivary gland field (Nam et al., 2019), and mini-pigs are becoming an increasingly popular model due to their comparative similarity to human anatomy (Wu et al., 2021).

## 5 Conclusion

Overall, this study has successfully proven that retinyl acetate can be incorporated into an electrospun fiber scaffold, which can successfully promote cell survival and proliferation in both normal and mimicked ROS environments whilst producing an antioxidant effect. This provides encouraging support for the potential use of such tissue engineering approaches to restoring the damaged microenvironment post-radiation to a state that can support the survival and proliferation of re-introduced cells, with the ultimate aim of tissue repair and ultimately restoration of salivary gland function. The work within this study emphasizes the potential of antioxidant tissue engineered scaffolds as a viable therapeutic approach and provides sufficient evidence to warrant further studies into such applications.

## Data Availability

The original contributions presented in the study are included in the article/Supplementary Material, further inquiries can be directed to the corresponding author.

## Supplementary material

SUPPLEMENTARY FIGURE S1 Cell viability results for TCP in both normal and H2O2 media.
