# Supplementary figures and images for: Fabrication of polycaprolactone electrospun fibres with retinyl acetate for antioxidant delivery in a ROS-mimicking environment
Source: Front Bioeng Biotechnol. 2023 Aug 15;11:1233801. doi: 10.3389/fbioe.2023.1233801 (PMC10463743; doi:10.3389/fbioe.2023.1233801)

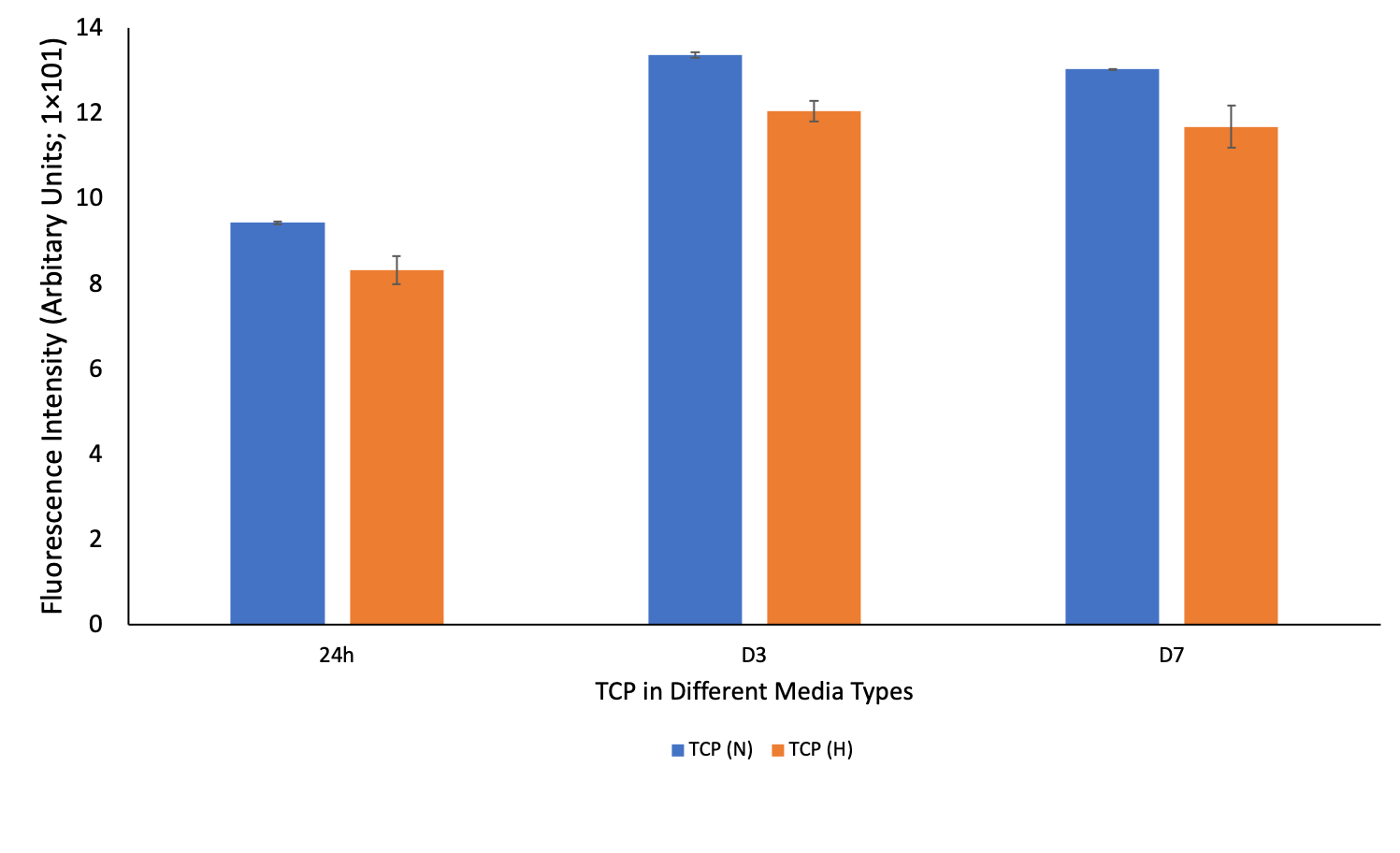

Supplement: Supplementary file 1 [file Image1.JPEG]
